# Prognostic and immunotherapeutic potential of regulatory T cell‐associated signature in ovarian cancer

**DOI:** 10.1111/jcmm.18248

**Published:** 2024-03-23

**Authors:** Yinglei Liu, Feng Shan, Ying Sun, Haili Kai, Yang Cao, Menghui Huang, Jinhui Liu, Pengpeng Zhang, Yanli Zheng

**Affiliations:** ^1^ Department of Obstetrics and Gynecology The Second Affiliated Hospital of Nantong University (First People's Hospital of Nantong City) Nantong China; ^2^ Department of Gynecology The First Affiliated Hospital of Nanjing Medical University Nanjing China; ^3^ Department of Lung Cancer Surgery Tianjin Lung Cancer Center, National Clinical Research Center for Cancer, Key Laboratory of Cancer Prevention and Therapy, Tianjin's Clinical Research Center for Cancer, Tianjin Medical University Cancer Institute and Hospital Tianjin China

**Keywords:** machine learning, ovarian cancer, regulatory T cell, tumour immune microenvironment

## Abstract

Tumour‐induced immunosuppressive microenvironments facilitate oncogenesis, with regulatory T cells (Tregs) serving as a crucial component. The significance of Treg‐associated genes within the context of ovarian cancer (OC) remains elucidated insufficiently. Utilizing single‐cell RNA sequencing (scRNA‐Seq) for the identification of Treg‐specific biomarkers, this investigation employed single‐sample gene set enrichment analysis (ssGSEA) for the derivation of a Treg signature score. Weighted gene co‐expression network analysis (WGCNA) facilitated the identification of Treg‐correlated genes. Machine learning algorithms were employed to determine an optimal prognostic model, subsequently exploring disparities across risk strata in terms of survival outcomes, immunological infiltration, pathway activation and responsiveness to immunotherapy. Through WGCNA, a cohort of 365 Treg‐associated genes was discerned, with 70 implicated in the prognostication of OC. A Tregs‐associated signature (TAS), synthesized from random survival forest (RSF) and Least Absolute Shrinkage and Selection Operator (LASSO) algorithms, exhibited robust predictive validity across both internal and external cohorts. Low TAS OC patients demonstrated superior survival outcomes, augmented by increased immunological cell infiltration, upregulated immune checkpoint expression, distinct pathway enrichment and differential response to immunotherapeutic interventions. The devised TAS proficiently prognosticates patient outcomes and delineates the immunological milieu within OC, offering a strategic instrument for the clinical stratification and selection of patients.

## 1 INTRODUCTION

Ovarian cancer (OC) is a fatal illness with a survival rate of only 30% over a 5‐year period, making it a top cause of cancer‐related deaths among females. Due to the absence of clear symptoms, it is typically identified at a later stage, further complicating its diagnosis. There are several subtypes of OC, each with different risk factors, genetic abnormalities and treatment responses.^1^ Recent advances in cancer research have led to the development of novel therapeutic approaches, including immunotherapy, targeted therapy and personalized medicine. In particular, immunotherapy has shown promising results in clinical trials, with some patients experiencing long‐lasting remission.^2^ Despite these advances, challenges remain in the early detection and effective treatment of OC.^3^ The identification of new biomarkers and the development of more sensitive and specific screening methods are critical to improving patient outcomes. Additionally, further research is needed to better understand the underlying biology of the disease and to identify new therapeutic targets.

In addition, OC is a highly immunosuppressive tumour, which can evade immune surveillance and resist anti‐tumour immunity. T lymphocytes play a vital part in the immune reaction to OC, as they are responsible for recognizing and eliminating tumour cells. However, OC is able to suppress T‐cell activation and proliferation, leading to an ineffective immune response. Recent studies have focused on understanding the complex interactions between OC cells and T cells, with the goal of identifying new therapeutic strategies to enhance T‐cell function and improve patient outcomes. For example, researchers have investigated the use of immune checkpoint inhibitors, which block inhibitory signalling pathways that prevent T cells from attacking tumour cells. In addition to immune checkpoint inhibitors, other promising immunotherapeutic approaches include chimeric antigen receptor (CAR) T‐cell therapy, T‐cell receptor (TCR) gene therapy and tumour‐infiltrating lymphocyte (TIL) therapy. These strategies aim to enhance T‐cell function and improve their ability to recognize and eliminate tumour cells.^4^ Regulatory T cells (Tregs) play a critical role in modulating immune responses and maintaining self‐tolerance. In the context of cancer, Tregs can exert both positive and negative effects on tumour growth and progression.^5^ On the one hand, Tregs can suppress anti‐tumour immune responses and promote tumour growth by inhibiting the activation and function of effector T cells. In addition, Tregs can promote angiogenesis and immunosuppressive cytokine production, further contributing to tumour growth and metastasis. On the other hand, Tregs may also have a beneficial role in cancer therapy. By suppressing excessive immune responses, Tregs can prevent autoimmunity and limit tissue damage caused by immune‐mediated cytotoxicity. This can be particularly important in immunotherapy approaches, such as immune checkpoint inhibitors and adoptive T‐cell therapy, which can result in immune‐related adverse events. The impact of Tregs on cancer prognosis and treatment response is complex and context‐dependent. While high levels of Tregs in the tumour microenvironment (TME) are generally associated with poor prognosis, Tregs may also play a beneficial role in limiting immune‐related toxicity in cancer therapy.^6^ Overall, the manipulation of Tregs in cancer therapy is an area of active research, with the goal of developing strategies to selectively target tumour‐promoting Tregs while preserving beneficial Treg function.

Artificial intelligence (AI) is a diverse set of technologies that aim to mimic human intelligence. Among these technologies, machine learning (ML) uses mathematical algorithms to detect patterns in data and make predictions. ML has shown remarkable effectiveness in various fields such as wireless communication, speech recognition and search engines.^7^ Evidence suggests that AI and ML can support healthcare providers in improving clinical diagnosis and treatment decisions, and may even replace human judgement in some cases.^8^ With genomics playing an increasingly prominent role in healthcare, it is anticipated that AI and ML will become essential tools for enabling precision oncology.

By integrating single‐cell and bulk RNA‐Seq data, we examined the infiltrating Treg component within OC. Employing weighted gene co‐expression network analysis (WGCNA) along with various machine learning approaches, we identified the optimal model for predicting the prognosis and immunotherapeutic response of OC patients.

## 2 METHODS

### 2.1 Data set acquisition and processing

We acquired the single‐cell RNA sequencing (scRNA‐Seq) data set GSE217517, encompassing eight OC samples, from the Gene Expression Omnibus (GEO, http://www.ncbi.nlm.nih.gov/geo/). In addition, microarray sequencing and clinical data were collated from data sets GSE140082 (*n* = 382), GSE14764 (*n* = 82), GSE17260 (*n* = 112) and GSE32062 (*n* = 262). To ensure comparability across data sets, expression data were converted to transcripts per million (TPM) format, and batch effects were mitigated using the ‘combat’ function within the ‘sva’ package.^9^, ^10^ From the Cancer Genome Atlas (TCGA) database, we retrieved bulk RNA‐Seq data, mutational profiles and clinical details pertaining to OC patients. Prior to analysis, all data sets underwent log2 transformation to standardize the format.

### 2.2 Processing flow of scRNA‐Seq data

The scRNA‐Seq data were processed using the ‘Seurat’ R package,^11^, ^12^ with cells exhibiting less than 200 features being excluded. The data set was normalized and scaled employing Seurat's NormalizeData and ScaleData functions, respectively, with batch effects mitigated via canonical correlation analysis (CCA).^13^ The ‘FindVariableFeatures’ function was utilized to identify the top 2000 variable genes. Clustering analysis was conducted using Seurat's *t*‐distributed stochastic neighbour embedding (t‐SNE) and the ‘FindClusters’ function at a resolution of 0.8.^14^ Marker genes were delineated using the ‘FindAllMarkers’ function within Seurat, selecting cluster‐specific markers. Cell classification leveraged common cellular markers, identifying differential genes within the Treg cell‐containing cluster as Treg‐specific marker genes. The ‘AUCell’ R package quantified gene set activity in individual cells, with gene expression rankings derived from the area under the curve (AUC) of the model genes. Cells exhibiting higher gene set expression levels corresponded to greater AUC values. The ‘AUCell exploreThresholds’ function determined the threshold for cells with active gene sets, and the ‘ggplot2’ R package visualized each cell's AUC score on a t‐SNE plot, facilitating the identification of active clusters.

### 2.3 Acquiring key genes for regulating tregs activity in bulk RNA‐Seq

In this investigation, single‐sample gene set enrichment analysis (ssGSEA)^15^, ^16^ was employed to calculate the enrichment percentage of specific gene sets within each sample, facilitating the assessment of Treg enrichment values in individual specimens from The Cancer Genome Atlas‐Ovarian Cancer (TCGA‐OC) data set. The WGCNA R package served as the methodological foundation for constructing gene co‐expression networks.^17^ Tumour samples were aggregated, and the optimal soft threshold for adjacency calculation was determined through visual inspection. Hierarchical clustering, guided by variances in the topological overlap matrix, allowed for the identification and amalgamation of related modules based on strong correlation values (*R* > 0.25). The module eigengene, representing the principal component of gene modules and serving as a surrogate for all genes within a specific module, was computed. Pearson correlation analysis was utilized to explore the association between module eigengene values and clinical traits. Genes within modules exhibiting the most significant correlations with the Treg score were selected for further analysis.

### 2.4 Tregs‐associated signature (TAS) produced using integrative machine learning methods

To develop a consensus signature with exceptional accuracy and stability, we harnessed a comprehensive suite of 10 machine learning algorithms. Our integrative approach encompassed a diverse array of methodologies, including Least Absolute Shrinkage and Selection Operator (Lasso), Elastic Net (Enet), Ridge Regression, Stepwise Cox Regression, CoxBoost, partial least squares Regression for Cox models (plsRcox), random survival forest (RSF), Super Predictor (SuperPC), generalized boosted regression models (GBM) and Survival Support Vector Machine (survival‐SVM). This signature was meticulously crafted to the 70 pivotal genes implicated in regulating Treg activity. Within the context of the TCGA‐OC cohort, TAS was constructed employing the Leave‐One‐Out Cross‐Validation (LOOCV) strategy.^18^ Subsequently, these models underwent rigorous validation across four additional data sets.

### 2.5 The construction of a nomogram

The clinical features were integrated with the TAS score to construct a more accurate nomogram using the ‘rms’ R package, thereby enhancing the ability to predict prognosis.^19^ We utilized calibration, c‐index and ROC curves to assess the performance of the nomogram.

### 2.6 Enrichment analysis and functional annotation

Using the ‘limma’ package,^20^ we did a differential gene expression analysis to compare high‐ and low‐TAS groups with a threshold of *p* < 0.05 and log2 (FC) > 1. We used the MSigDB database to choose ‘the c2.cp.kegg.v7.4.symbols.gmt’ as the gene set of interest. We used the R packages ‘clusterProfiler’ and ‘org.Hs.eg.db’ to perform GSEA and Gene Ontology (GO) enrichment for the differentially expressed genes. The ‘ggplot2’ and ‘GseaVis’ R programmes were then used to illustrate the results of the enrichment study visually.

### 2.7 Mutation analysis

To see the somatic mutations in the OC low‐ and high‐TAS groups, we used the maftools R package. Using information from the TCGA database, the mutation annotation format (MAF) was created. For each OC patient, we estimated the tumour mutation burden (TMB).

### 2.8 Immunotherapy and evaluation of immune microenvironment

We obtained the data of OC patients from the TIMER 2.0 database and evaluated immune infiltration using 7 different methods in the TCGA database. Heat maps were used to display variations in immune cell infiltration across different TAS groups. Differences and correlations in immune checkpoint genes were examined using boxplots and scatter plots between high‐ and low‐TAS groups. Using ssGSEA, the enrichment scores of 29 immune signatures were quantified. The immune scores, stromal scores and ESTIMATE scores of OC patients were calculated by utilizing the ‘estimate’ R package.^21^ To predict immunotherapy sensitivity, the Cancer Immunome Atlas (TCIA) database was mined for Immunophenoscores (IPS) for TCGA‐OC. In this study, we compared IPS in high‐TAS and low‐TAS populations.^22^

### 2.9 Statistical analysis

All statistical analyses and data processing tasks were conducted using R version 4.1.3. Survival analysis was performed utilizing Kaplan–Meier curves, with the log‐rank test applied to determine statistical significance. The R package ‘survminer’ was employed for the generation of all survival curves. Heatmaps were created using the ‘pheatmap’ R package. For variables following a normal distribution, quantitative differences were assessed using either a two‐tailed *t*‐test or one‐way analysis of variance (ANOVA). Non‐normally distributed data were analysed employing the Wilcoxon test or the Kruskal–Wallis test. All statistical procedures were executed within the *R* environment, considering a *p*‐value <0.05 as the threshold for statistical significance.

## 3 RESULTS

### 3.1 Identification of Treg characteristic genes

**FIGURE 1:**
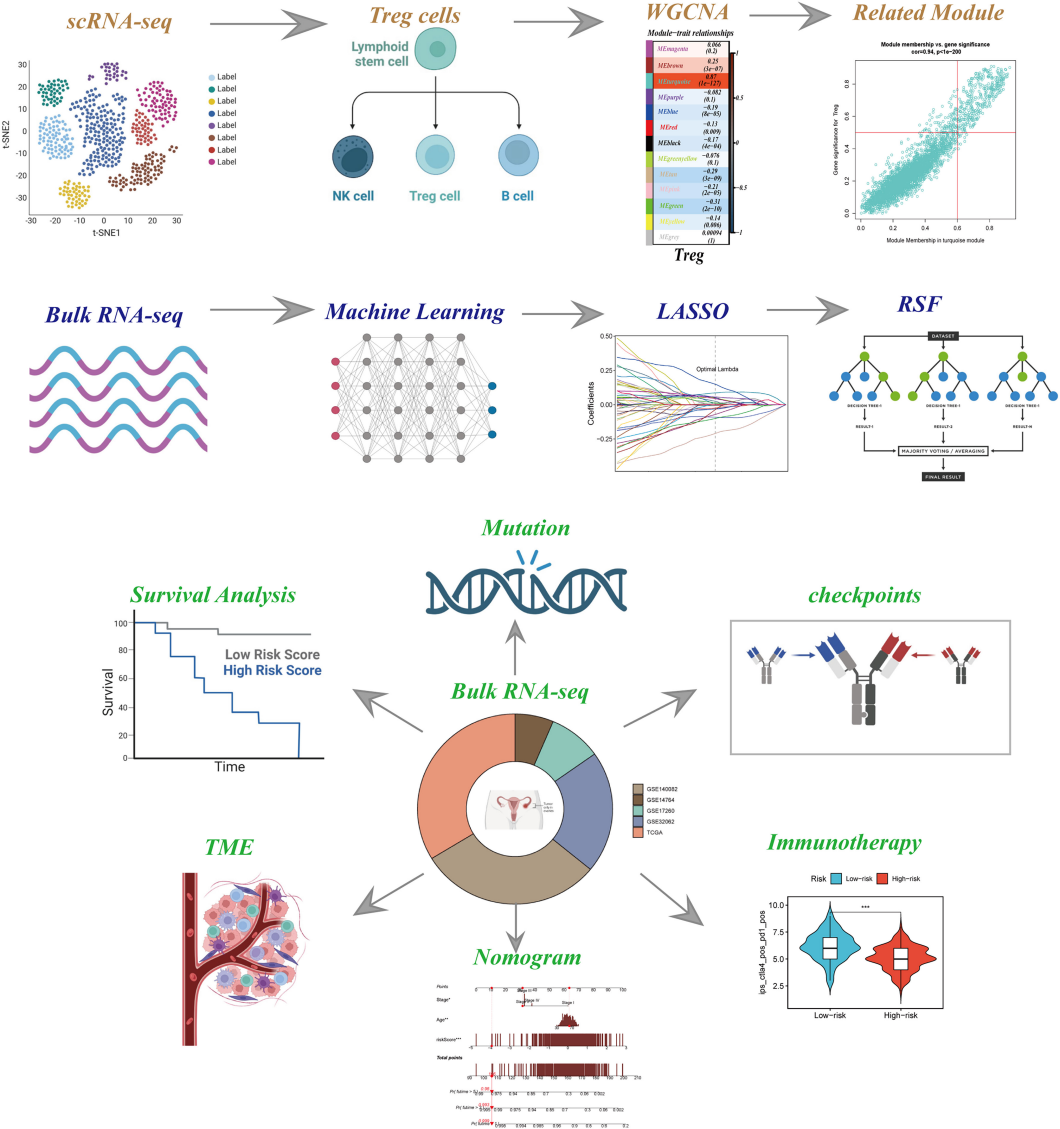
Investigational flowchart.

**FIGURE 2:**
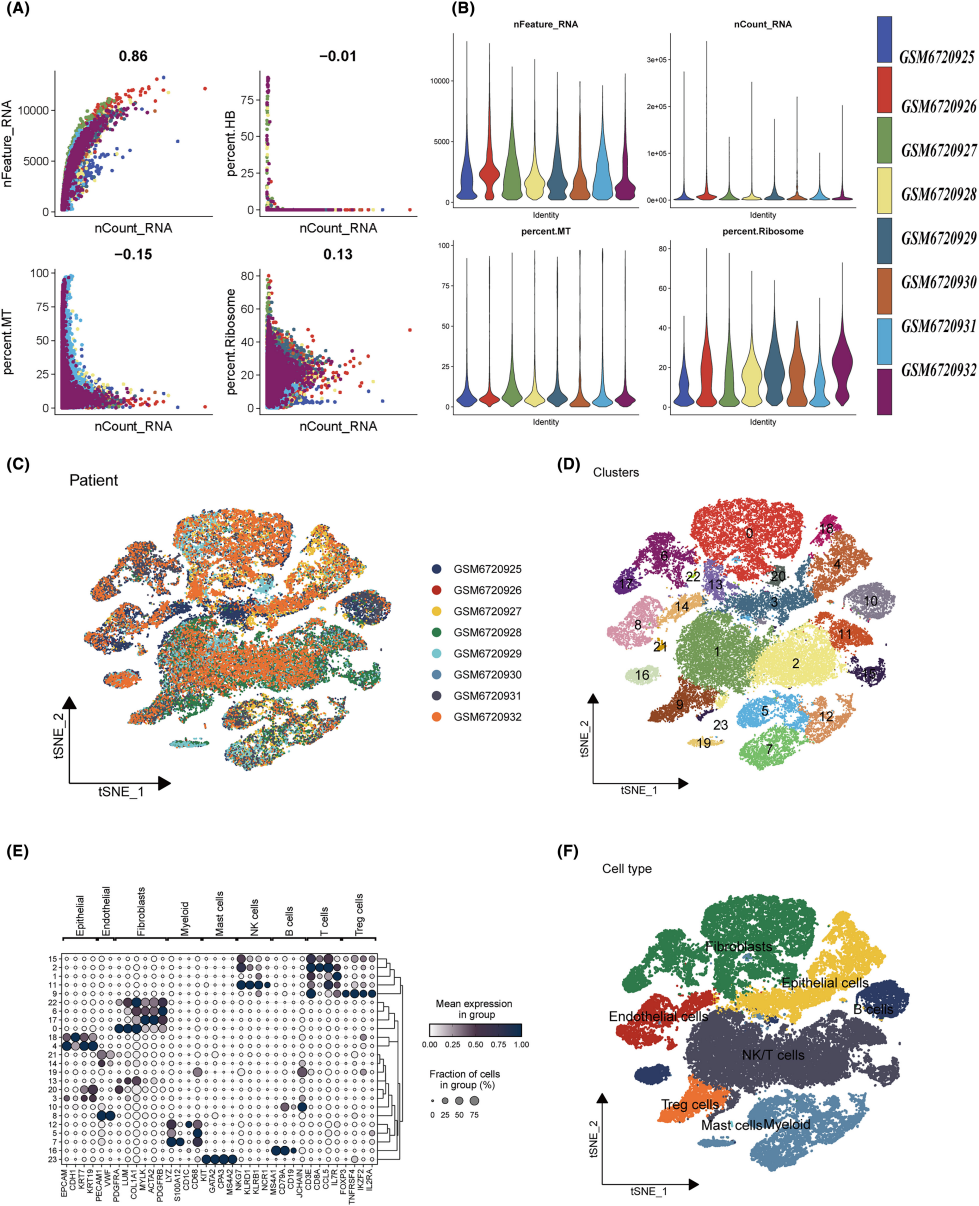
Single‐cell data annotation and regulatory T‐cell (Treg) extraction. (A) Analysis of the correlation between sequencing depth and gene expression levels, including percentages of mitochondrial genes, red blood cell genes and ribosomal genes. (B) Distribution of gene expression levels, sequencing depth and percentages of red blood cell, mitochondrial and ribosomal genes across eight samples. (C) Cell distribution across eight samples visualized via t‐SNE plots. (D) t‐SNE plot classification of cells in eight ovarian cancer (OC) samples into 24 clusters. (E) Bubble plot representation of characteristic marker genes for each cell cluster. (F) t‐SNE visualization indicating the annotation of OC samples into seven cell types within the tumour microenvironment (TME), with different colours representing distinct cell types.

Figure 1 depicted the study's flow diagram. The correlation between sequencing depth and gene expression levels, the percentage of mitochondrial genes, the proportion of genes associated with red blood cells, and the percentage of ribosome genes was depicted in Figure 2A. The distribution of gene expression levels, sequencing depth, the percentage of red blood cell genes, the proportion of genes related to mitochondria and the percentage of ribosome genes in the eight samples were illustrated in Figure 2B. The gene expression profiles of 52,420 cells from eight OC samples were acquired for further analysis after data processing and filtering. The consistency of cell distribution within each sample suggested that no noticeable batch effect existed across samples, which may prove beneficial for future studies (Figure 2C). Afterwards, all cells were classified into 24 clusters using t‐SNE as the dimensionality reduction method (Figure 2D). The marker genes verified and recognized by many researchers are displayed in the bubble plot, and the expression levels of marker genes used by different clusters can be seen, and the type of cells of the cluster can be inferred (Figure 2E). The cell identification for each cluster was obtained by comparing DEGs with canonical marker genes, and cells in Cluster 9 were classified as Tregs (Figure 2F). Therefore, we identified 258 Tregs marker genes. Supplementary Figure S1 used the AUCell algorithm to calculate the AUCell score based on the marker of the difference table of Tregs, and shows the changes of AUCell score in each cell type by t‐SNE plot.

### 3.2 Identifying the Tregs‐associated signature (TAS)

**FIGURE 3:**
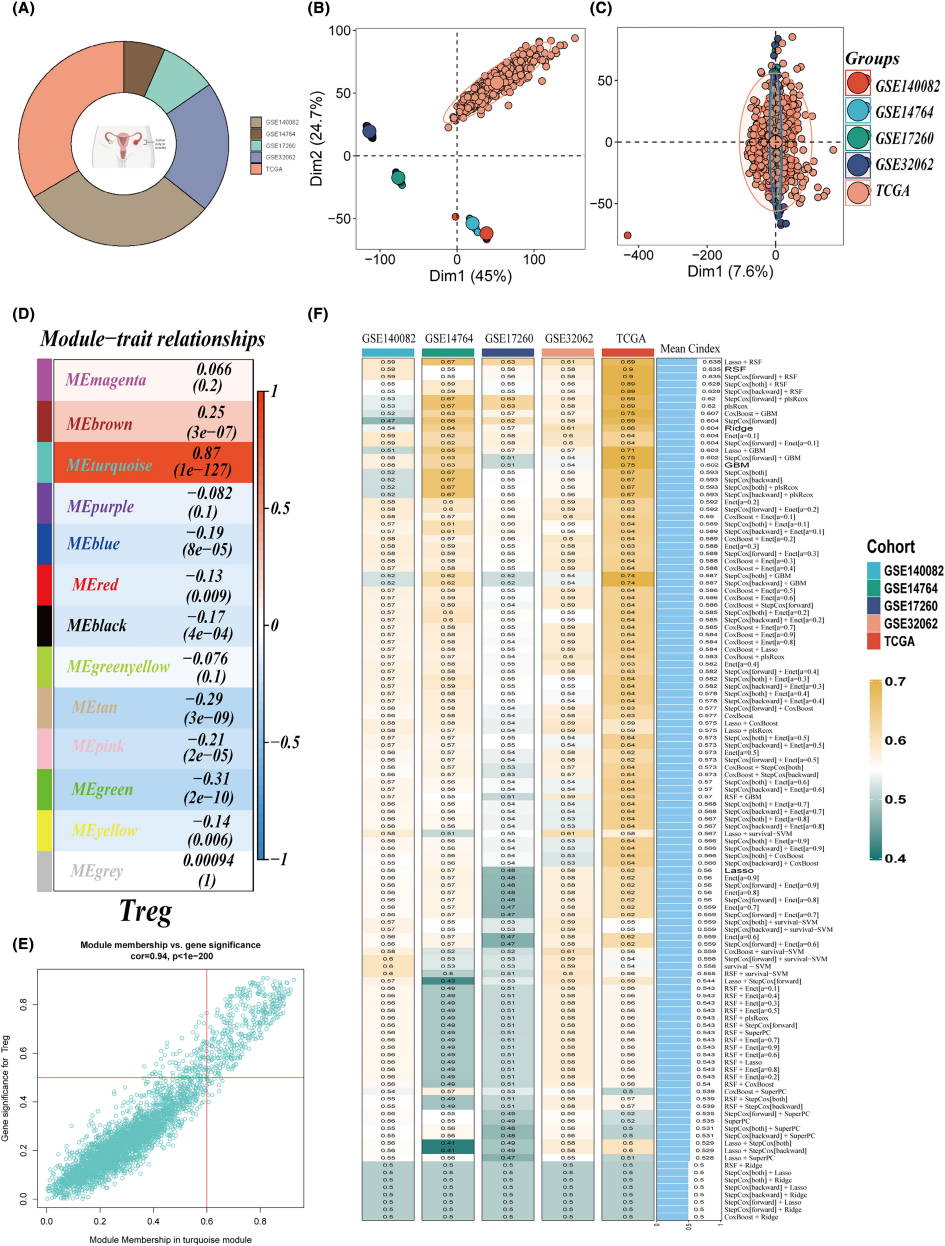
Signature development. (A) Analysis of sample sources and sample size proportions across five data sets. (B, C) Principal component analysis (PCA) plots before and after batch effect removal for five data sets. (D) Weighted gene co‐expression network analysis (WGCNA) identified modules closely associated with Tregs activity. (E) High correlation between gene significance and module membership in the turquoise module (correlation = 0.94). (F) Evaluation of 117 prediction models and calculation of the concordance index (C‐index) for each model across all validation data sets.

To enhance comparability between TCGA and GEO data sets, batch effects across the five data sets were mitigated. Figure 3A graphically presents the sample proportions within the five data sets. Figure 3B,C depict the sample distributions before and after batch effect removal, illustrating a uniform distribution post‐removal, signifying the efficacy of the batch normalization process. Utilizing differential expression markers of Tregs, TCGA‐OC samples were assessed using the ssGSEA algorithm, with the WGCNA algorithm subsequently employed to identify gene modules most indicative of Treg activity scores. These modules were deemed critical in regulating Treg scores. Setting the soft threshold value to 3 optimized mean connectivity within a power‐law distribution. Criteria set for module identification included a minimum of 100 genes per module and a similarity threshold >0.25 for module merging, resulting in 13 distinct non‐grey gene modules. The correlation of each gene module with clinical features was examined, revealing the turquoise module as most closely associated with Treg scores (Figure 3D). A significant positive correlation (*R* = 0.94) between gene significance and module membership underscored the high quality of gene module construction (Figure 3E). Univariate Cox analysis identified 70 genes linked to prognosis, from which a consensus TAS was developed through machine learning‐integrated approaches. Employing the LOOCV method, 117 predictive models were fitted to the TCGA‐OS data set, with model efficacy gauged by the c‐index in the validation data set (Figure 3F). Notably, a model combining Lasso and RSF algorithms emerged superior, achieving the highest average C‐index (0.638) across all validation data sets. The Lasso algorithm pinpointed four genes of paramount importance, while the RSF algorithm computed a TAS score.

### 3.3 Evaluating model stability

**FIGURE 4:**
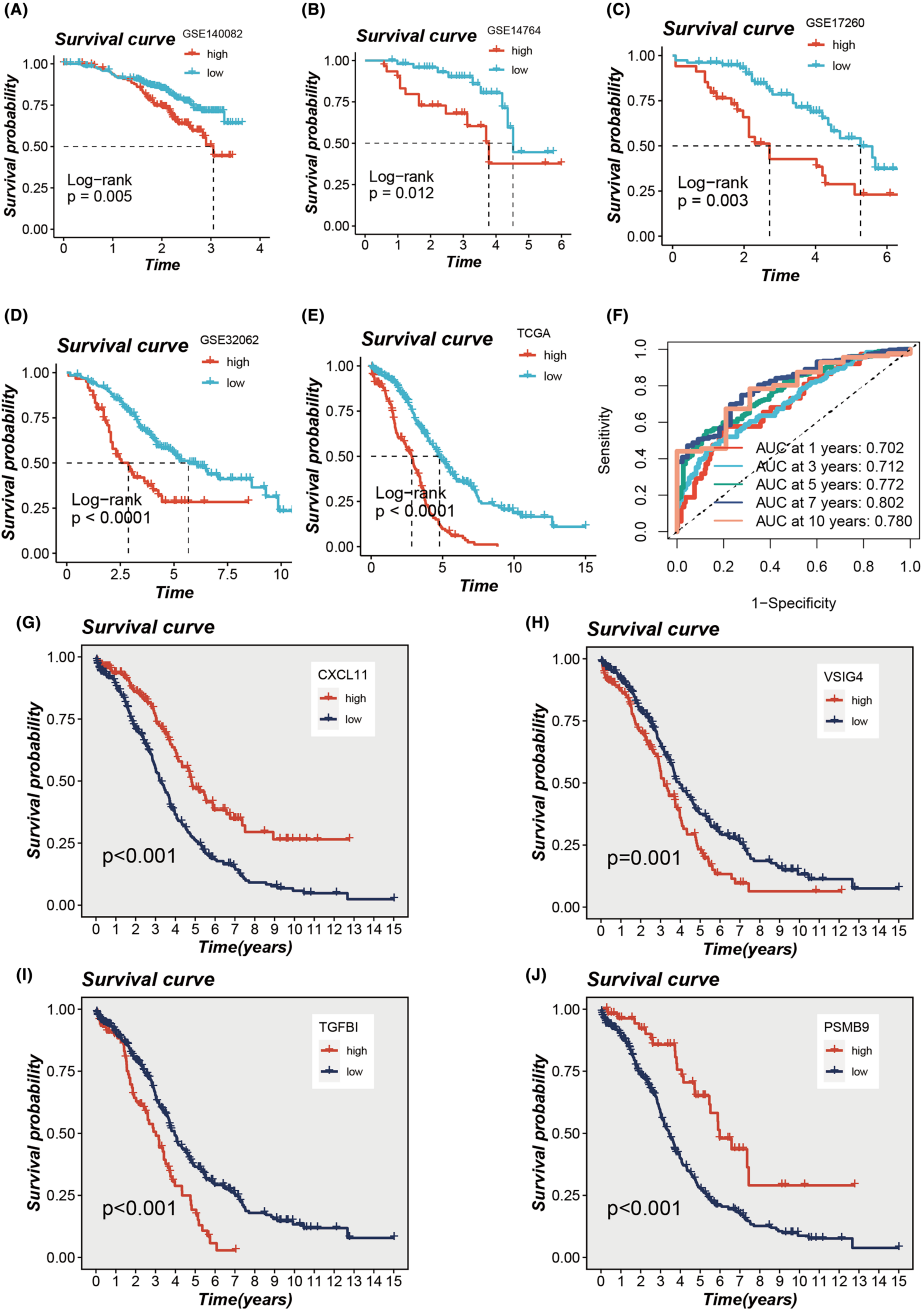
Assessment of TAS. (A–E) Kaplan–Meier survival analysis for signatures within The Cancer Genome Atlas (TCGA) and four GEO data sets. (F) Receiver operating characteristic (ROC) curve analysis of model performance in the TCGA data set. (G–J) Influence of four model genes on survival outcomes for OC patients in the TCGA cohort.

Patients were stratified into high‐ and low‐TAS groups based on the median TAS score. The high‐TAS group exhibited poorer prognoses, a trend consistent across all five cohorts (*p* < 0.05, Figure 4A–E). The model's performance was rigorously evaluated through ROC curves, with Figure 4F displaying the predictive accuracy of TAS scores within the TCGA cohort for forecasting OC patient outcomes at 1‐, 3‐, 5‐, 7‐ and 10‐year intervals, achieving AUC values exceeding 0.7. Furthermore, the prognostic relevance of the four genes central to the model was scrutinized. It was discerned that elevated expression levels of CXCL11 and PSMB9 were associated with favourable prognoses, whereas increased expression of VSIG4 and TGFBI correlated with adverse outcomes (Figure 4G–J).

### 3.4 Enrichment analysis

**FIGURE 5:**
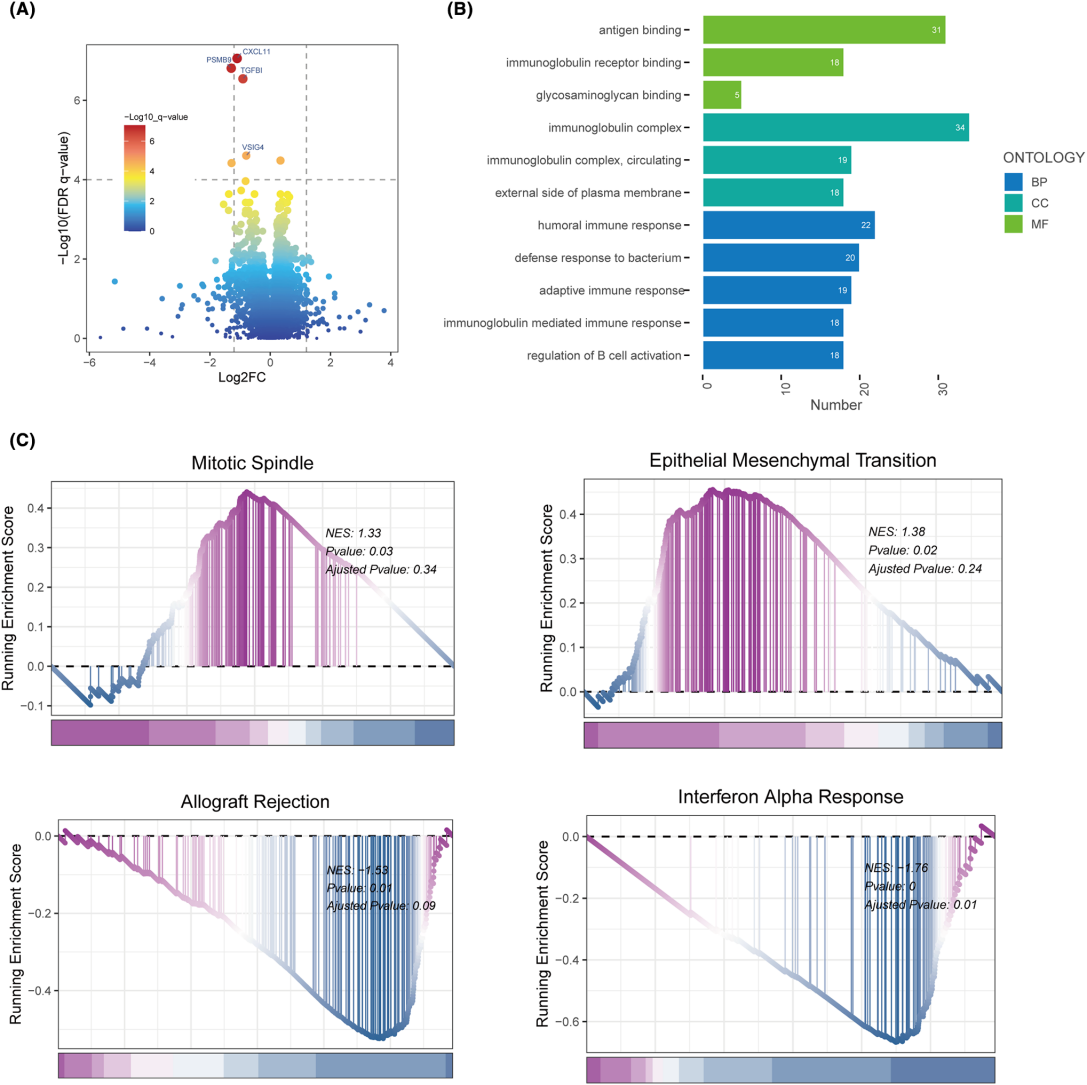
Enrichment analysis and functional annotation. (A) Identification of differentially expressed genes between high‐ and low‐TAS groups (FDR <0.05, log2(Fold Change) >1). (B) GO enrichment analysis presented via bar graphs. (C) Gene set enrichment analysis (GSEA) highlighted pathway distinctions between high‐ and low‐TAS groups.

Leveraging the limma package, differential gene expression between high‐ and low‐TAS groups was quantified and subsequently visualized in a volcano plot, with model genes specifically highlighted within the diagram (Figure 5A). To elucidate the pathway enrichment disparities between the TAS groups, GO enrichment analysis was performed on the differential genes. This analysis uncovered that the top three enriched biological process pathways included humoral immune response, defence response to bacteria and adaptive immune response. Within cellular components, the foremost enriched pathways were the immunoglobulin complex, circulating immunoglobulin complex and the external side of the plasma membrane. Pertaining to molecular functions, the principal enriched pathways encompassed antigen binding, immunoglobulin receptor binding and glycosaminoglycan binding (Figure 5B). Additionally, GSEA was employed to further investigate the pathway enrichment differences between the high‐ and low‐TAS groups. The analysis revealed that the high‐TAS group predominantly exhibited enrichment in pathways such as mitotic spin (normalized enrichment score (NES) = 1.33, *p* = 0.03) and epithelial–mesenchymal transition (EMT, NES = 1.38, *p* = 0.02). Conversely, the low‐TAS group showed significant enrichment in allograft rejection (NES = −1.53, *p* = 0.01) and interferon‐α response (NES = −1.76, *p* = 0.00) (Figure 5C). Existing research highlights the EMT pathway as a critical mechanism for cancer cell proliferation and metastasis.^23^ Consequently, disease progression in the high‐TAS group may be furthered by the activation of the EMT pathway, thereby portending a poorer prognosis for affected patients.

### 3.5 Building a nomogram with better predictive performance

**FIGURE 6:**
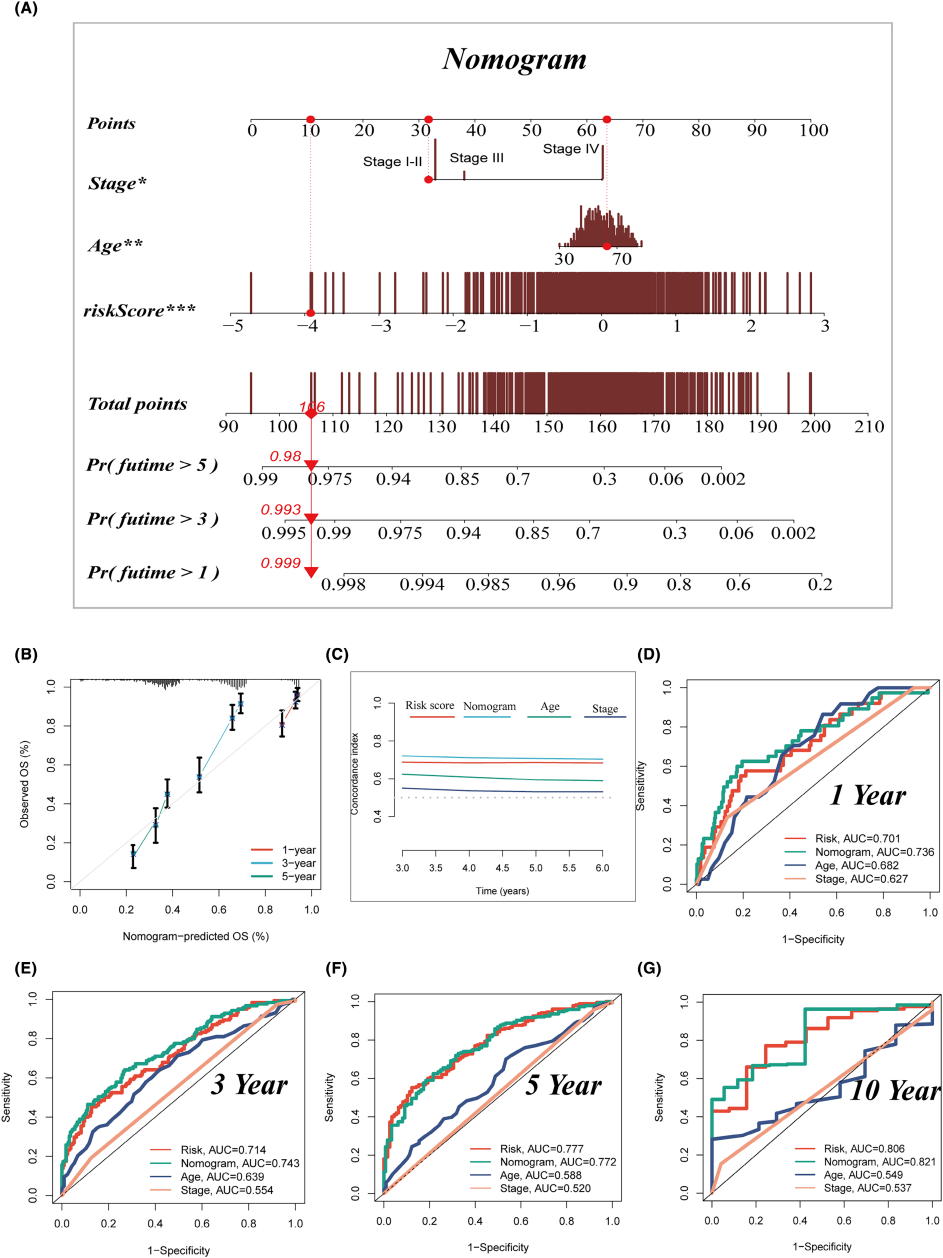
Enhanced nomogram accuracy. (A) Construction of a nomogram by integrating clinical features with TAS groups. (B) Calibration curves for the nomogram at 1‐, 3‐ and 5‐year predictions. (C) Concordance index (C‐index) curves assessed predictive performance of various clinical parameters, nomogram scores and TAS scores. (D–G) ROC curves for 1‐, 3‐, 5‐ and 10‐year forecasts presented area under curve (AUC) values for clinical factors, TAS scores and nomogram scores.

Integrating patient clinical stage, age and TAS score, we developed a nomogram to enhance the accuracy of prognosis prediction for OC patients, thereby augmenting model performance (Figure 6A). To ascertain the nomogram's effectiveness, calibration curves were generated (Figure 6B), showcasing precise prognostic predictions for OC patients at 1, 3 and 5 years. Moreover, the concordance index (C‐index) curves underscored that our nomogram scores outperformed traditional clinical features and TAS scores in predictive capability (Figure 6C). The prognostic accuracy of our nomogram was further validated through ROC analysis, evidencing its superior performance compared to conventional clinical features and TAS scores. The AUC values at 1, 3, 5 and 10 years were 0.736, 0.743, 0.772 and 0.821, respectively (Figure 6D–G). These results affirm the nomogram's efficacy in prognosticating outcomes for OC patients, illustrating its advantage over existing clinical features and TAS scores.

### 3.6 Mutation landscape

**FIGURE 7:**
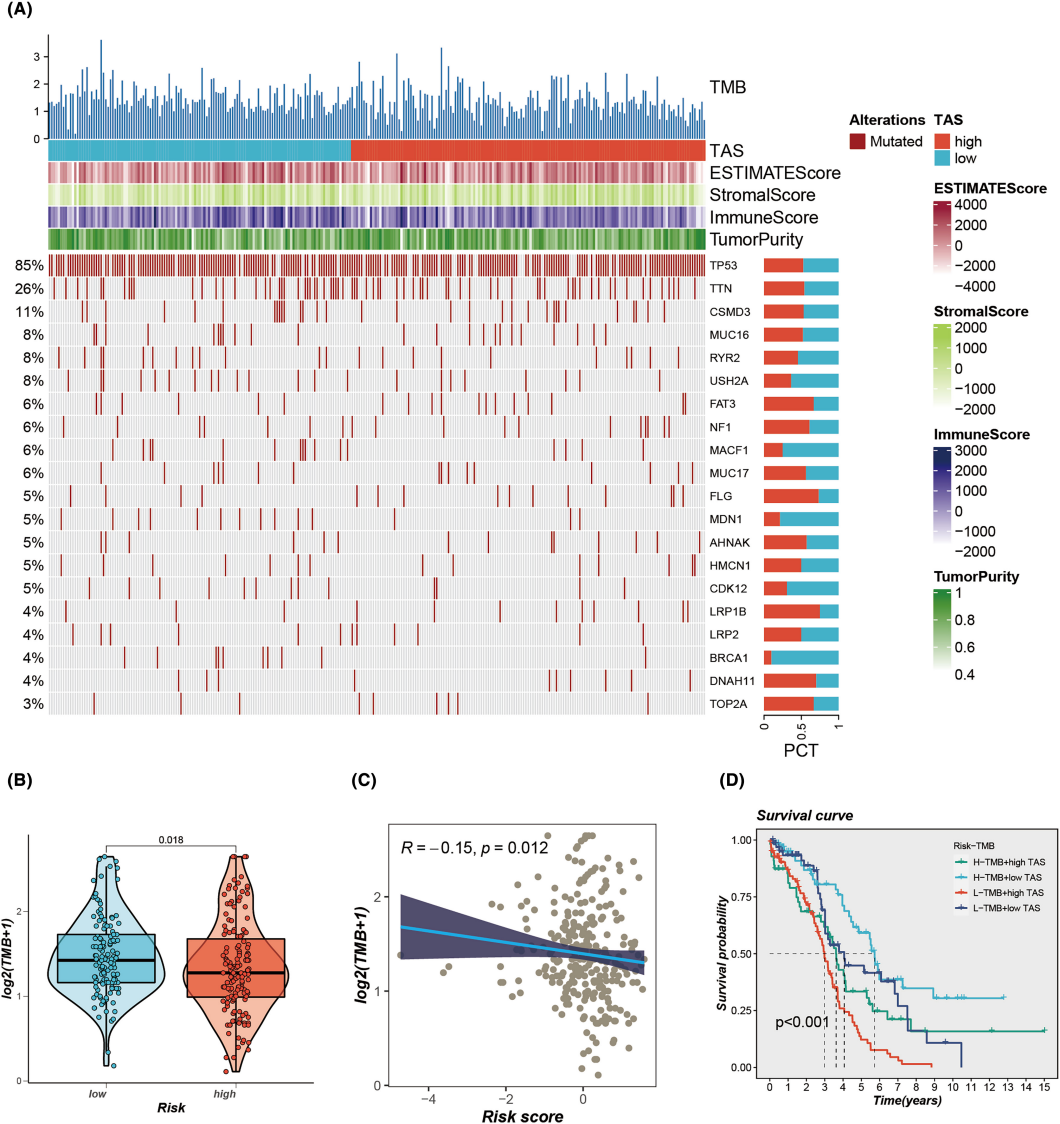
Mutation analysis. (A) Mutation landscape for the top 20 frequently mutated genes in high‐ versus low‐TAS groups. (B) Tumour mutational burden (TMB) differences between high‐ and low‐TAS patients. (C) Correlation between TAS score and TMB. (D) Survival analysis across four groups: high‐TMB/high‐TAS, high‐TMB/low‐TAS, low‐TMB/high‐TAS and low‐TMB/low‐TAS.

The analysis of somatic mutations within the TCGA‐OC cohort elucidated that the diversity of these mutations has significant implications for cancer immunotherapy outcomes. Figure 7A displays the mutation profile identified in this study. Additionally, a comparison of TMB between high‐ and low‐TAS groups revealed that the mutation frequency in OC was comparatively higher in the low‐TAS group (Figure 7B). A significant positive correlation between TAS score and TMB was established through Spearman correlation analysis (*R* = −0.15, *p* = 0.012, Figure 7C). Patients were categorized into four segments based on the median TMB value and median TAS score: high‐mutation + high‐TAS, high‐mutation + low‐TAS, low‐mutation + high‐TAS and low‐mutation + low‐TAS. The outcomes demonstrated that individuals with high‐mutation and low‐TAS OC exhibited the most favourable prognosis, whereas those with low‐mutation and high‐TAS OC experienced the least favourable outcomes (Figure 7D).

### 3.7 Assessment of immune microenvironment

**FIGURE 8:**
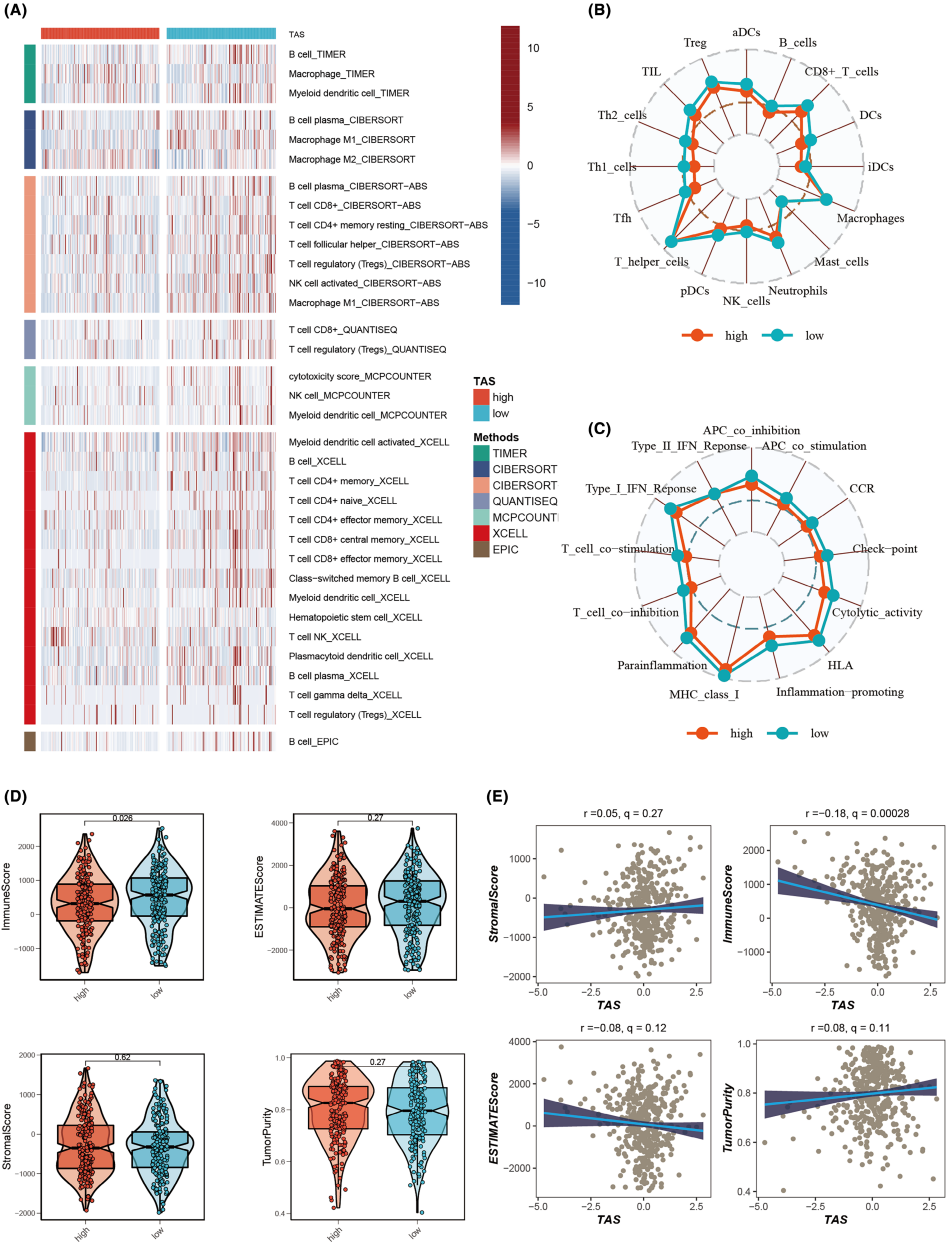
Immune cell content and immune checkpoint analysis. (A) Assessment of immune cell content differences between TAS groups using seven distinct algorithms. (B, C) Quantification of immune cell infiltration and immune function between high‐TAS and low‐TAS groups via the ssGSEA algorithm. (D) Violin plot illustrating the difference in TME Score calculated using the ESTIMATE algorithm between TAS groups. (E) Spearman's correlation analysis of TAS Score and TME Score.

**FIGURE 9:**
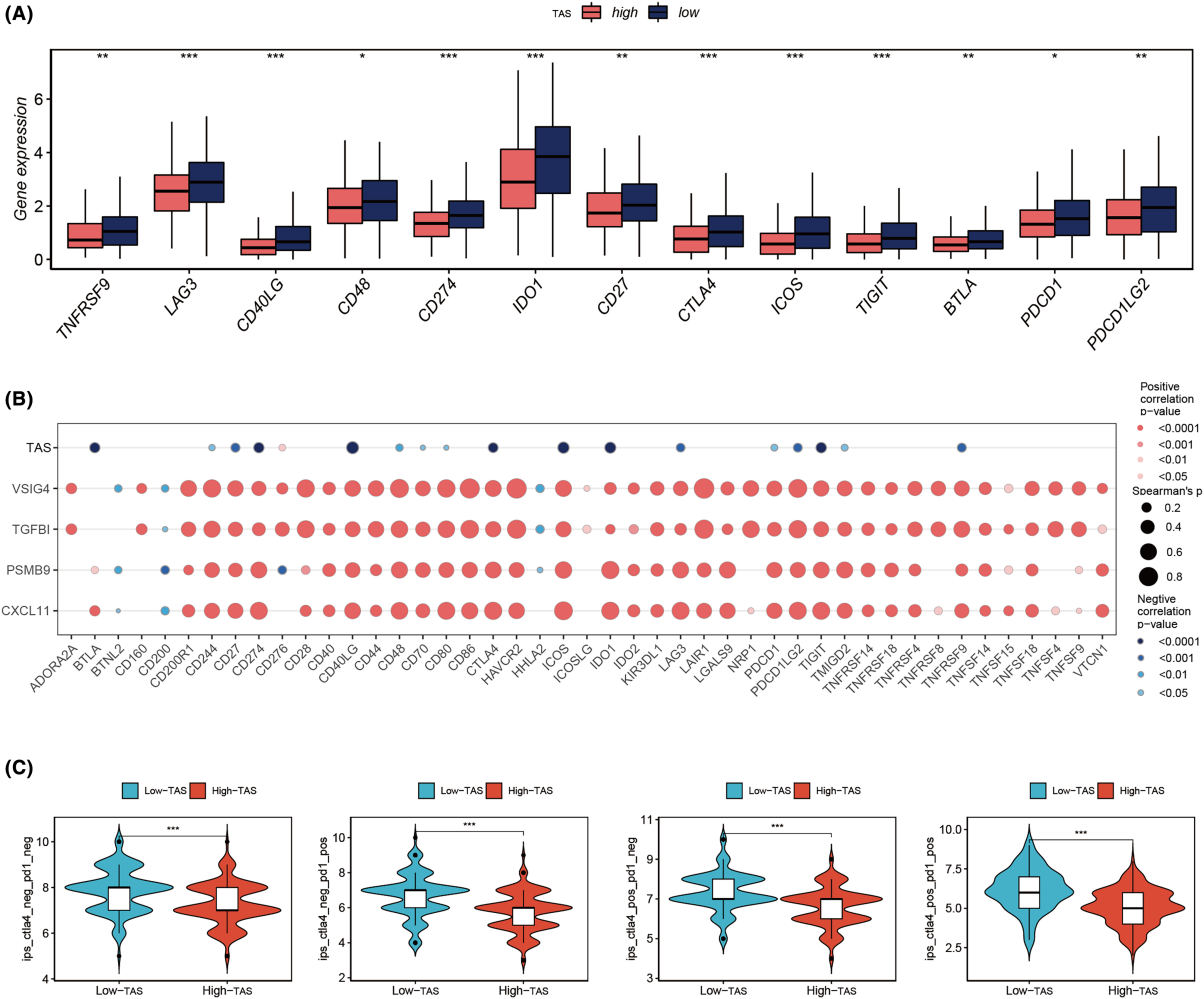
Immune checkpoint and The Cancer Immunome Database (TCIA) analysis. (A) Box plot depicting differences in immune checkpoint gene expression between high‐ and low‐TAS groups. (B) Correlation analysis between model genes and immune checkpoints. (C) Significantly higher immune phenotype scores (IPS) in various categories within the low‐TAS group. *p < 0.05, **p < 0.01, ***p < 0.001.

This investigation utilized data from seven immune infiltration algorithms in the TIMER database to evaluate immune infiltration disparities between high‐ and low‐TAS groups in the TCGA‐OC cohort. The findings indicated enhanced immune infiltration levels within the low‐TAS group (Figure 8A). Employing the ssGSEA algorithm, further analysis on immune cell infiltration and function between the TAS groups was conducted. Notably, OC patients in the low‐TAS group demonstrated elevated infiltration of various immune cells, including active dendritic cells (ADC), B cells, CD^8^+ T cells, dendritic cells (DCs), immature dendritic cells (iDC), neutrophils, NK cells, plasmacytoid dendritic cells (pDCs), helper T cells (Th^1^/Th^2^), tumour‐infiltrating lymphocytes (TILs) and Tregs (Figure 8B,C). Additionally, the low‐TAS group presented higher levels of APC co‐inhibition, APC co‐stimulation, chemokine receptor (CCR), checkpoint, cytolytic activity, HLA, inflammation‐promoting, MHC Class I, parainflammation, T‐cell co‐inhibition, T‐cell co‐stimulation and Type I IFN responses (Figure 8B,C). Through the ESTIMATE method, the study validated immune infiltration levels across different TAS groups. Spearman correlation analysis revealed a negative correlation between TAS score and immune score, suggesting that the degree of immune cell infiltration in the TME might account for the differences in disease progression and the efficacy of immunotherapy in OC patients (Figure 8D,E). Furthermore, the study examined the association between TAS scores and well‐known immune checkpoints in the TCGA‐OC cohort, with the low‐TAS group exhibiting elevated expression of nearly all immune checkpoint genes (ICG), such as PD‐1, PDL‐1 and CTLA4 (Figure 9A). The correlation between model genes, TAS scores and ICGs was analysed and depicted in bubble plots (Figure 9B). Intriguingly, expression levels of model genes were positively correlated with most immune checkpoints, whereas TAS scores were negatively correlated with the expression levels of certain common immune checkpoints, including PD‐L1, CD40LG, IDO1, LAG3 and TIGIT. This observation provides crucial insights into the potential of immune checkpoint blockade therapy for OC patients. Conclusively, the study assessed the immune phenotype score (IPS) across different TAS groups to discern patients who might derive greater benefit from immunotherapy. The analysis showed that the low‐TAS group had significantly higher IPS scores, suggesting a pronounced benefit from this type of immunotherapy (Figure 9C).

## 4 DISCUSSION

Previous investigations looked into the connection between immune cell populations and cancer patients’ clinical outcomes.^24^ The TME in different cancer types consists of diverse immune cell populations that contribute to a complex immunological network. Despite the presence of tumour‐reactive T cells, CD^8^ and CD^4^ T cells in cancer patients often remain inactive, leading to tumour evasion. The immunosuppressive effects of Treg, which inhibit the function of effector T cells, have been linked to poorer outcomes in OC patients. Treg infiltration in the TME and peripheral blood of OC patients suggests their involvement in the development and progression of OC, highlighting the potential for Treg targeting as a therapy for OC. To achieve this, understanding the functions of Treg‐related genes is crucial in identifying potential targets for therapy. Knowledge of the molecular mechanisms underlying Treg‐mediated immunosuppression could facilitate the development of more effective treatments for OC.^25^

In this investigation, scRNA‐Seq was utilized to examine eight OC specimens, leading to the delineation of seven unique cellular phenotypes. The Treg population was identified through their characteristic marker genes, followed by a differential gene analysis that yielded 258 Treg‐associated genes. WGCNA was applied to score OC samples in bulk RNA‐Seq data sets, pinpointing module genes critically related to Treg scores. A total of 70 genes associated with Tregs and their prognostic significance were subsequently identified. The pivotal genes regulating Treg activity were explored, and an integrative approach was adopted to formulate a consensus TAS. 117 distinct models were fitted to the training data set, with a combination of the Lasso and RSF algorithms demonstrating optimal efficacy. Prognostic evaluations indicated that individuals within the high‐TAS group faced an adverse prognosis. The TAS showcased notable precision and consistent performance across four additional public GEO data sets, as evidenced by ROC analysis. Clinical data integration led to the development of a nomogram designed to improve the predictive accuracy of the study. The nomogram scores outperformed conventional TAS scores and other clinical parameters in forecasting patient survival outcomes.

Numerous pathway enrichment analyses were performed to elucidate the mechanisms underlying the survival differences observed between the high‐ and low‐TAS groups. The appropriate organization of the mitotic spindle is essential for preserving genetic stability, and abnormalities in this process may promote tumour formation and advancement. Some findings emphasized the critical function of PI3K‐C2α in the organization of the mitotic spindle and its potential as a target for cancer therapy.^26^ The high‐TAS group in OC is characterized by a significant enrichment of the mitotic spindle signature, which can have a substantial impact on the occurrence and progression of the disease by regulating mitotic spindle pathway activity. The biological process of EMT plays a crucial role in tumour progression, enabling epithelial cells to acquire a mesenchymal phenotype, and promoting self‐renewal, resistance to apoptosis and chemotherapy. Various factors, including deregulated oncogenic signalling pathways, hypoxia and cells present in TME, can initiate and promote EMT. As a result, EMT leads to the loss of epithelial cell polarity and cell–cell adhesion, while promoting enhanced invasive and migratory properties.^27^ We can speculate that the increased EMT activity in the high‐TAS group promotes the progression of OC, thus making the prognosis of high‐TAS patients worse.

Previous research studies have established a significant statistical association between TMB and the FIGO stage, grade and residual tumour size in patients with OC. Specifically, a higher TMB has been linked to more favourable clinical outcomes in OC patients.^28^ In past studies, it has been observed that TMB is positively associated with the infiltration of immune cells and the elicitation of an inflammatory T‐cell‐mediated response in different cancer types. Consequently, a higher TMB may enhance the sensitivity of tumours to immunotherapy.^29^ Increased TMB has been associated with a heightened immune response and increased infiltration of CD8‐positive and PD‐L1‐positive T cells in various cancers, making it a valuable biomarker for predicting the efficacy of immunotherapy. However, in this study, patients with high‐TAS OC were found to have more frequent mutations in genes, while those with low‐TAS had lower TMB levels. Based on TMB levels, patients were categorized into four groups, with the high‐mutation + low‐TAS group exhibiting the most favourable prognosis. These findings provide new insights into evaluating patient prognosis and may have clinical implications for personalized treatment approaches.

A diverse array of components, such as the extracellular matrix, tumour‐infiltrating lymphocytes and neoangiogenesis, constituted the TME. The TME played a critical role in the efficacy of cancer immunotherapy, and precise interventions in the TME could lead to the in vivo activation of cytotoxic T cells for tumour elimination.^30^ The study aimed to determine the significance of immune infiltration in tumours by comparing the immune cell infiltration between high‐ and low‐TAS groups. The results showed that low‐TAS tumours had higher immune cell infiltration as indicated by the immune infiltration algorithm, and this was further validated by the ESTIMATE algorithm. The immune score was negatively correlated with the TAS score, indicating that the low‐TAS group had significantly higher immune cell infiltration. Furthermore, we analysed the expression of immune checkpoints, such as PD‐L^1^ and CTLA‐4, in different TAS groups. The low‐TAS group had higher expression of immune checkpoints, and most immune checkpoint genes were negatively correlated with the TAS score. To assess the potential differences in immunotherapy response between the TAS groups, we used The Cancer Immunome Database (TCIA) to study the effects of PD‐1 and CTLA‐4 treatment. The IPS score of OC patients in the low‐TAS group was significantly higher than that in the high‐TAS group, indicating that patients in the TAS‐risk group could potentially benefit more from immunotherapy.

This study had several limitations that should be acknowledged. First, the TAS was developed based on existing data sets, and further validation in large‐scale prospective clinical studies is necessary to confirm the findings. Additionally, while our results suggest that the signature could serve as a prognostic biomarker and predict immunotherapy response, more research is needed to validate these findings. However, our use of integrated analysis and machine learning provided valuable insights into the prognostic significance of Tregs in OC and its potential therapeutic implications.

## AUTHOR CONTRIBUTIONS

**Yinglei Liu:** Supervision (equal); validation (equal). **Feng Shan:** Methodology (equal); visualization (equal). **Ying Sun:** Investigation (equal); writing – original draft (equal). **Haili Kai:** Project administration (equal); validation (equal). **Yang Cao:** Supervision (equal). **Menghui Huang:** Formal analysis (equal); validation (equal). **Jinhui Liu:** Data curation (equal); formal analysis (equal); visualization (equal). **Pengpeng Zhang:** Conceptualization (equal); data curation (equal). **Yanli Zheng:** Data curation (equal); supervision (equal).

## CONFLICT OF INTEREST STATEMENT

The authors declare that the research was conducted in the absence of any commercial or financial relationships that could be construed as a potential conflict of interest.

## CONSENT FOR PUBLICATION

All authors consent to the publication of this study.

## Supporting information


**Supplementary Figure S1.** AUCell scores were calculated for each cell based on Tregs differentially expressed marker genes, and divided into two groups of high and low expression based on the median value of the scores.

## Data Availability

The data sets analysed in the current study are available in the TCGA repository (http://cancergenome.nih.gov/), and GEO (https://www. ncbi.nlm.nih.gov/geo/).
