# Supplementary figures and images for: Prognostic and immunotherapeutic potential of regulatory T cell‐associated signature in ovarian cancer
Source: J Cell Mol Med. 2024 Mar 23;28(8):e18248. doi: 10.1111/jcmm.18248 (PMC10960174; doi:10.1111/jcmm.18248)

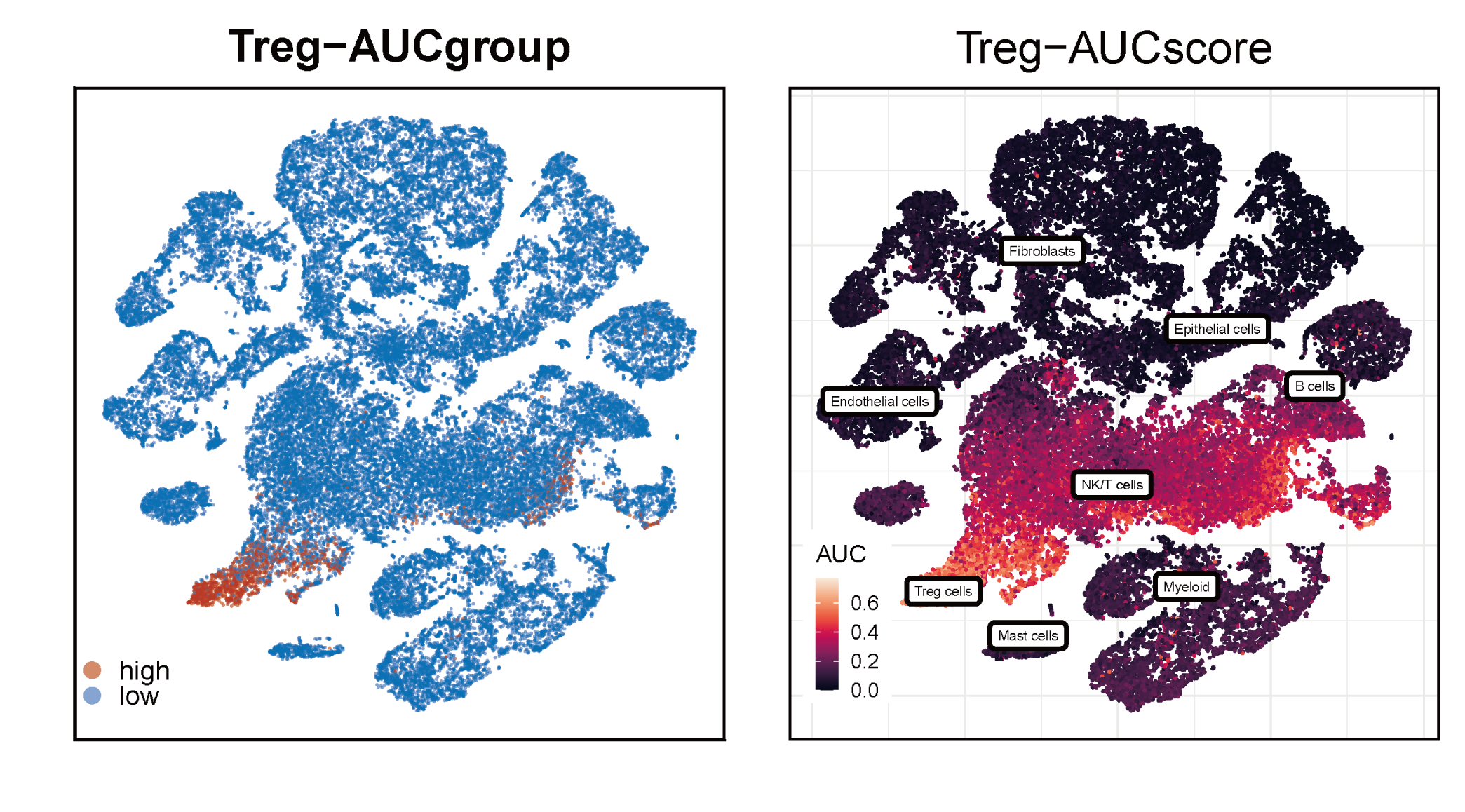

Supplement: Supplementary file 1 — Supplementary Figure S1. AUCell scores were calculated for each cell based on Tregs differentially expressed marker genes, and divided into two groups of high and low expression based on the median value of the scores. [file JCMM-28-e18248-s001.tif]
